# Supplementary figures and images for: Cholesterol binding to the sterol-sensing region of Niemann Pick C1 protein confines dynamics of its N-terminal domain
Source: PLoS Comput Biol. 2020 Oct 6;16(10):e1007554. doi: 10.1371/journal.pcbi.1007554 (PMC7537887; doi:10.1371/journal.pcbi.1007554)

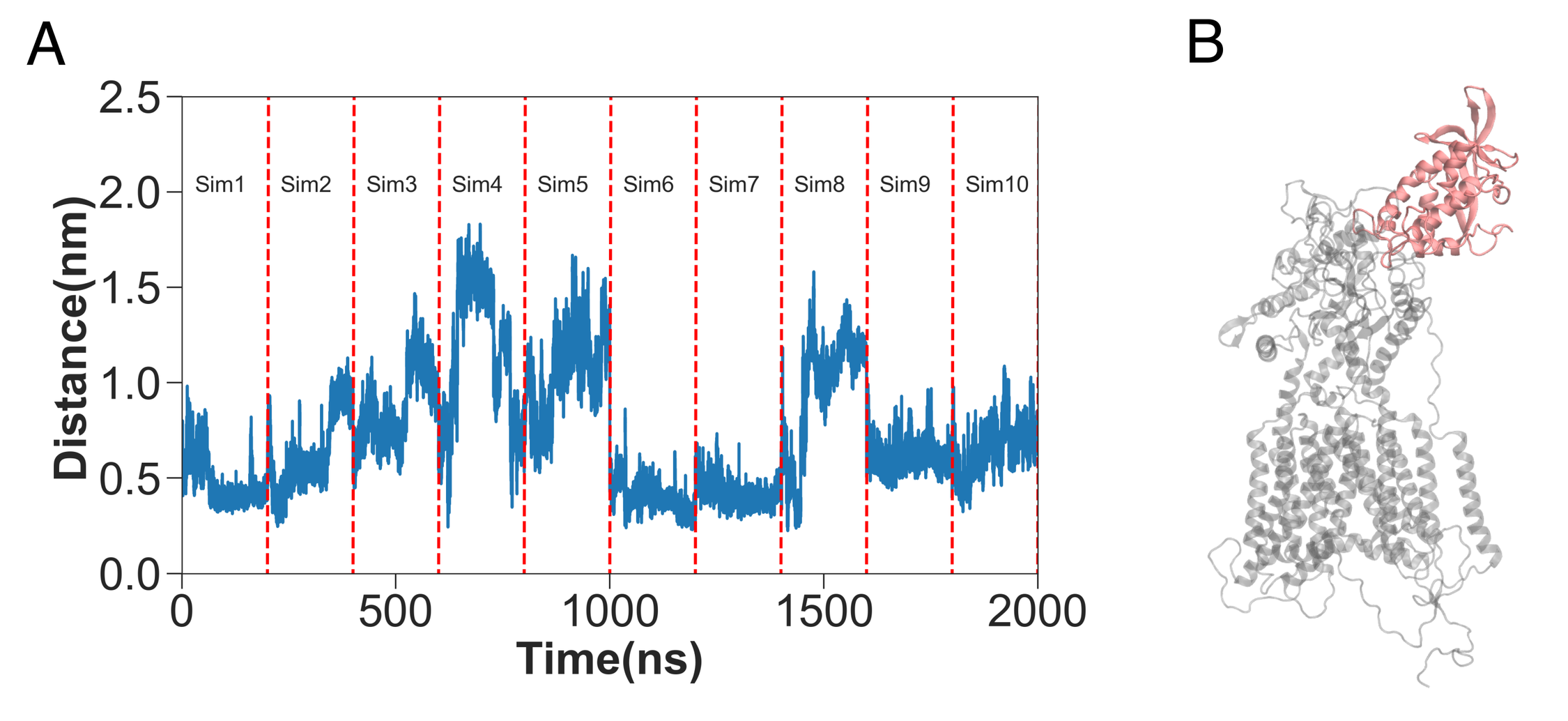

Supplement: S1 Fig — (A) The distance between cholesterol and the centre of mass of the cholesterol-binding residues in the binding site as shown in Fig 1, for the 10 different simulation replicas of the POPC-CHOL-bound simulations. Cholesterol temporarily escapes the binding pocket in replicas 3,4,5 and 8. However, in replicas 3 and 4, cholesterol returns to the binding site within 200 ns. (B) Simulation snapshot from sim4, showing that the NTD tilts when cholesterol leaves the SSD binding site. (TIF) [file pcbi.1007554.s001.tif]

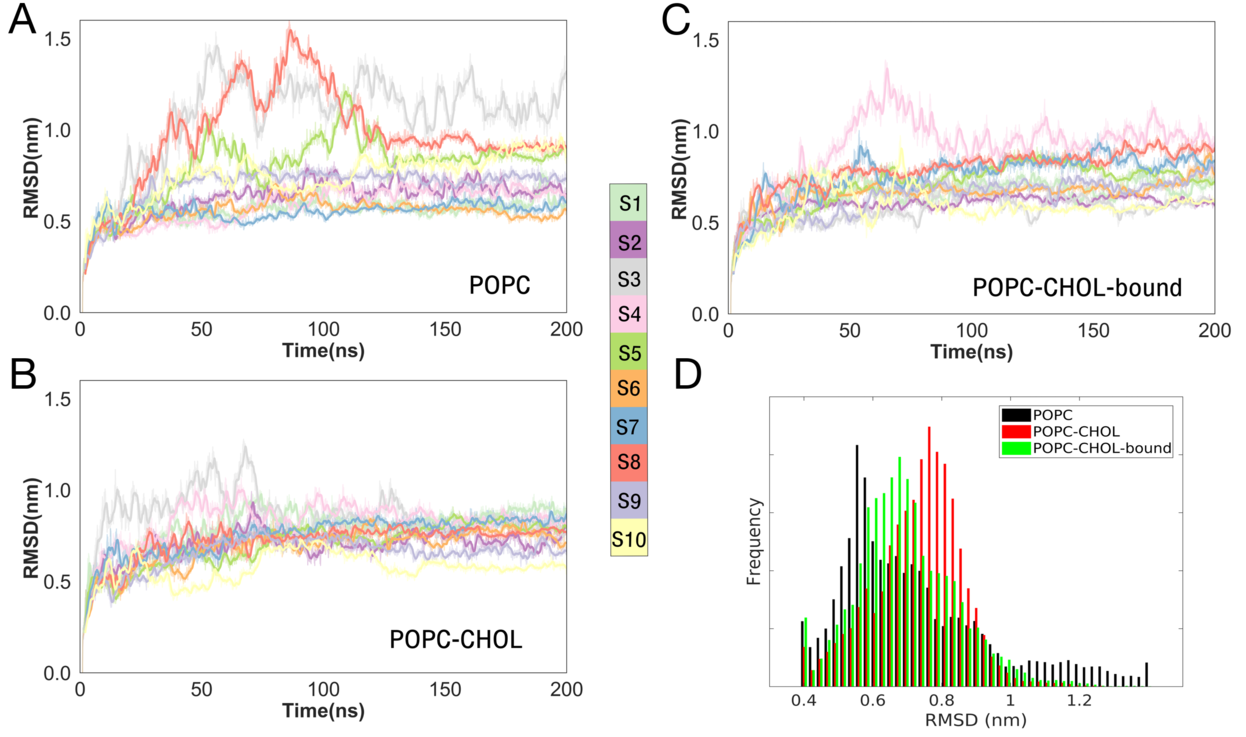

Supplement: S2 Fig — (A), (B) and (C) Root mean squared deviation (RMSD) from the initial structure for all simulations. S1 through S10 denote the 10 different simulation replicates. (D) Histogram of the RMSD. (TIF) [file pcbi.1007554.s002.tif]

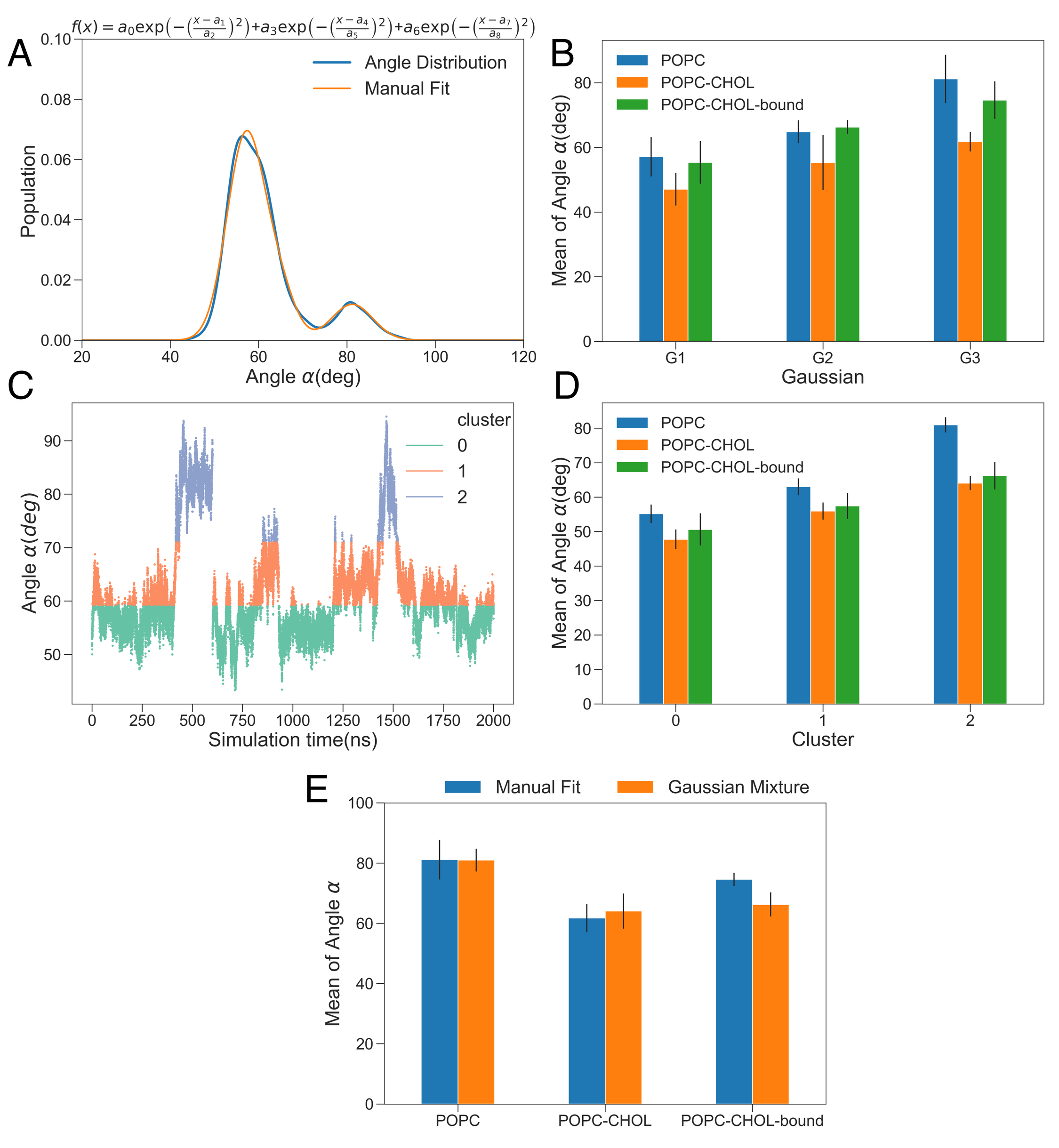

Supplement: S3 Fig — Two approaches were used: manual fitting and fitting using a Gaussian mixture model. (A) Manual fitting using the sum of 3 Gaussians. Initial conditions for the fitting were chosen by inspecting the distribution. The function and fit were refined by using the Akaike Information Criterion/Bayesian Information Criterion (BIC). (B) Means and standard deviations of each Gaussian obtained from manual fitting. (C) Clusters obtained from the Gaussian Mixture model mapped onto the angle α time series. (D) Means and standard deviations of the clusters. (E) Comparison of the results for cluster 2 and Gaussian 3 (i.e. representatives of the flexed state) obtained from manual fitting and Gaussian Mixture model. (TIF) [file pcbi.1007554.s003.tif]

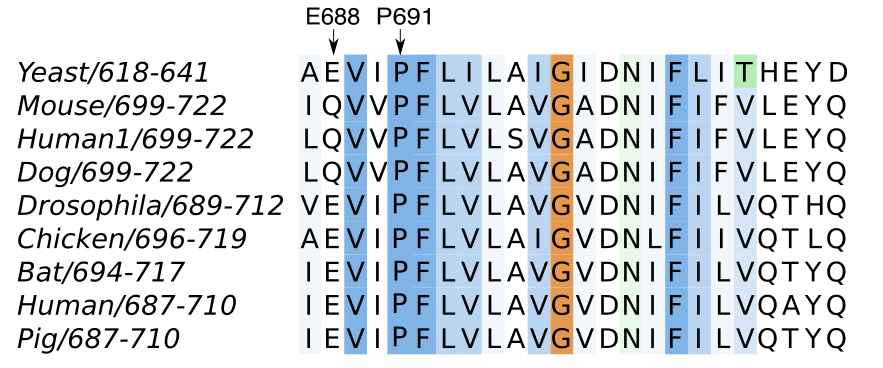

Supplement: S4 Fig — The different colours in the alignment correspond to conservation of different types of residues. (TIF) [file pcbi.1007554.s004.tif]

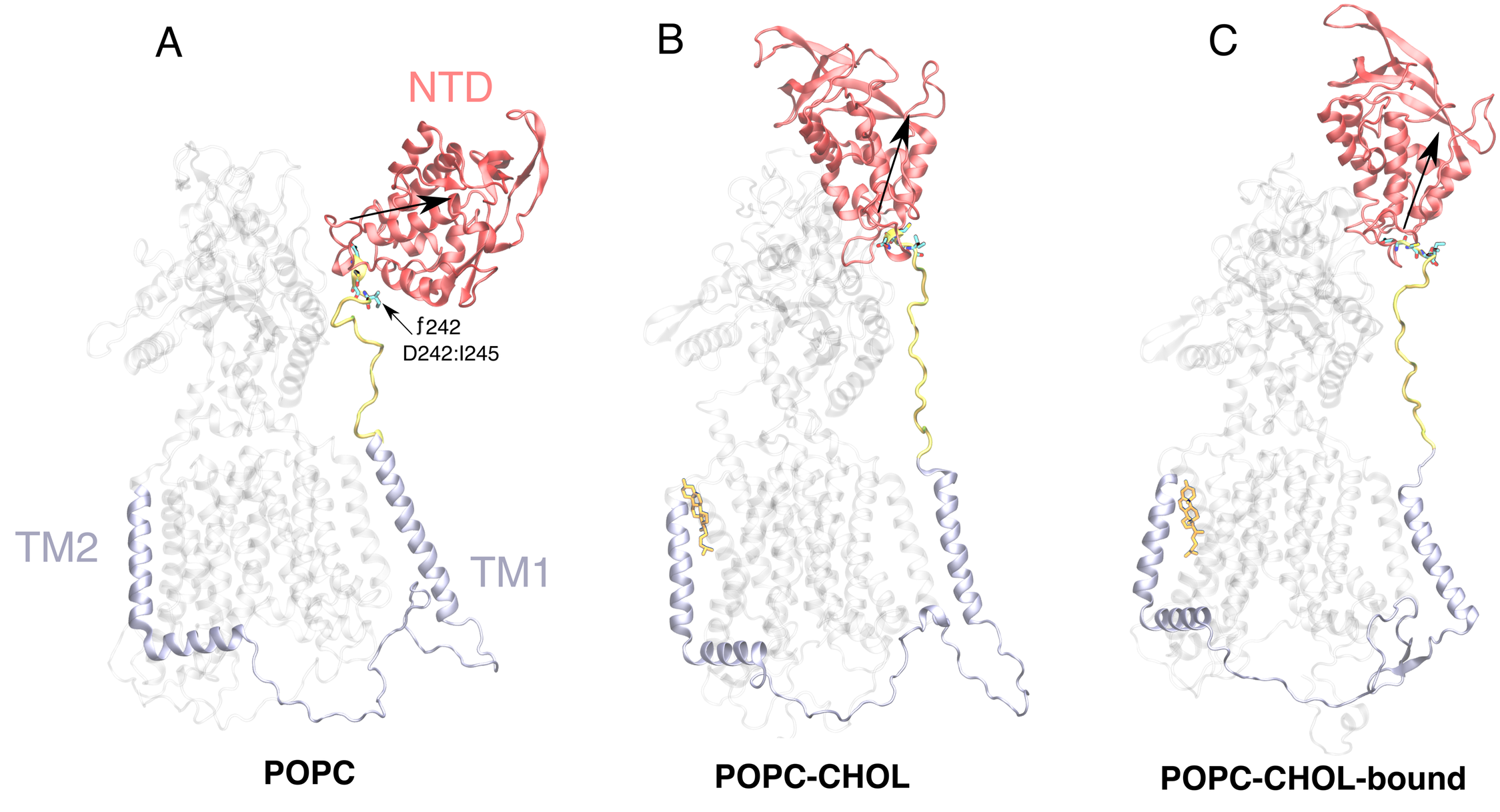

Supplement: S5 Fig — The black arrow shows the putative entry point of a sterol in the NTD [18]. (TIF) [file pcbi.1007554.s005.tif]

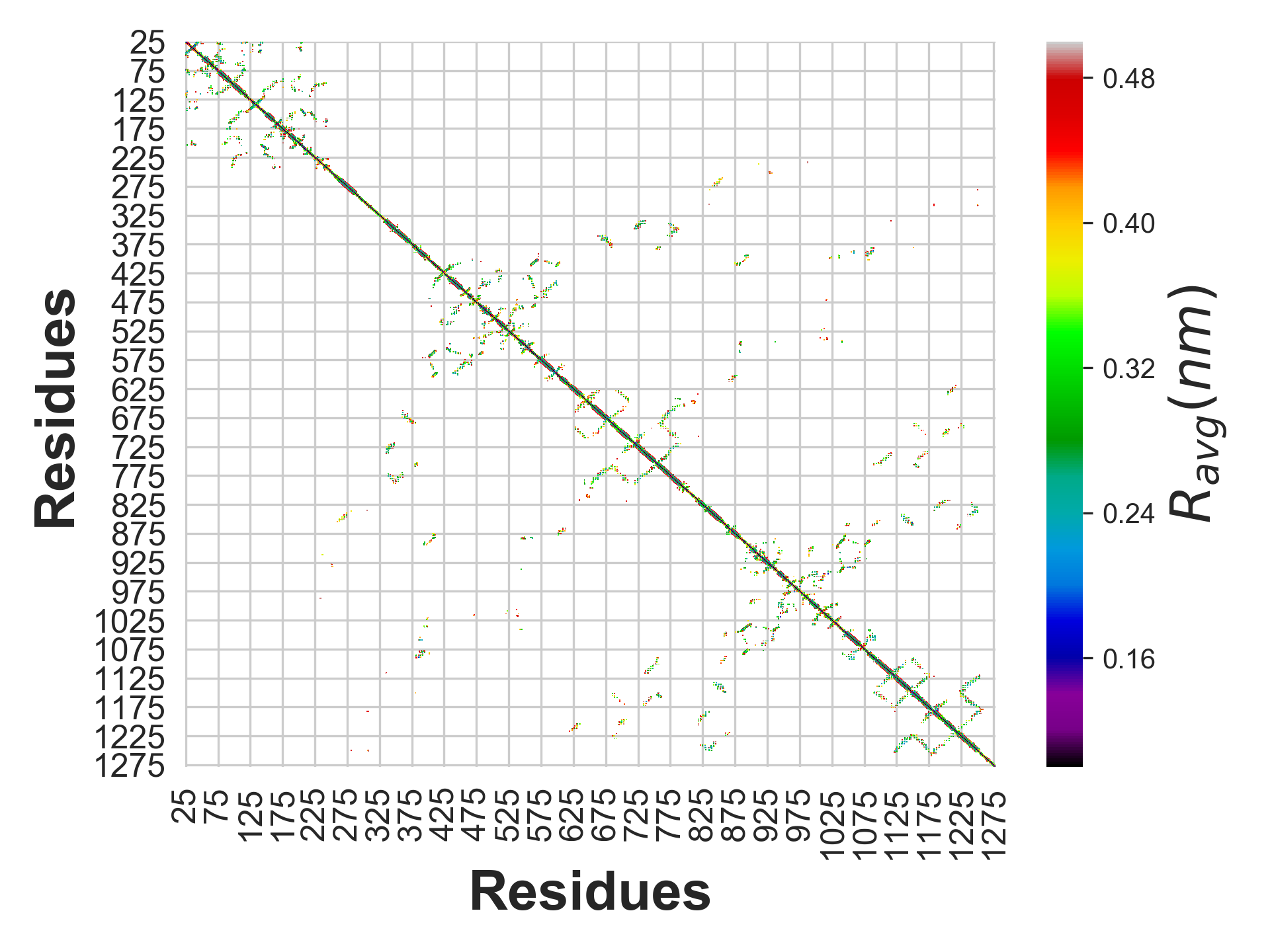

Supplement: S6 Fig — The matrix is obtained by calculating average distances between residue pairs. Only residue pairs within a cut-off distance of 0.5 nm are shown. (TIF) [file pcbi.1007554.s006.tif]

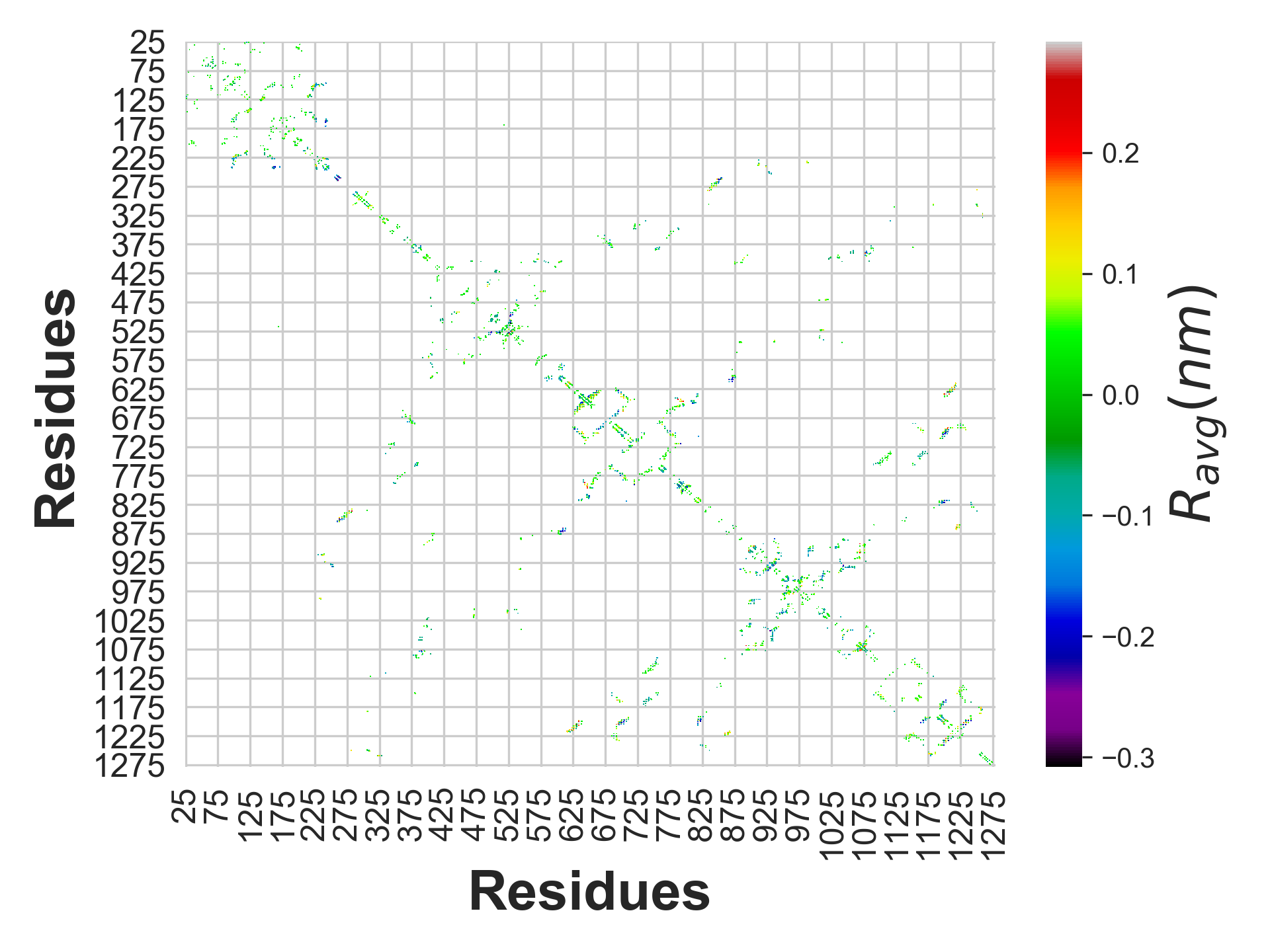

Supplement: S7 Fig — The matrix is obtained by subtracting average distances of the POPC-CHOL simulations from the average distances of the POPC simulations. (TIF) [file pcbi.1007554.s007.tif]

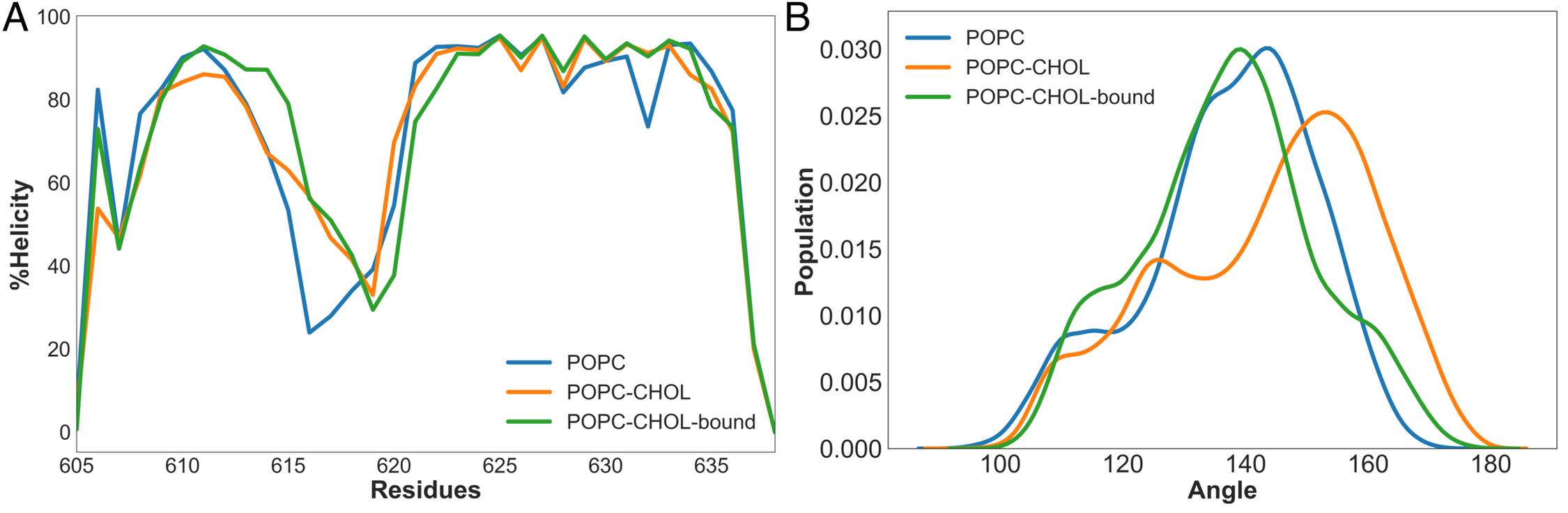

Supplement: S8 Fig — (A) Percentage helical content for TM3 compared for all three sets of simulations. (B) Distribution of the angle between TM3a (residues Asp620 to Leu639 and TM3b (residues Thr604 to Arg615) for all 10 POPC, POPC-CHOL and POPC-CHOL-bound simulations. The difference between the distributions is not as apparent here as it is in Fig 7. (TIF) [file pcbi.1007554.s008.tif]

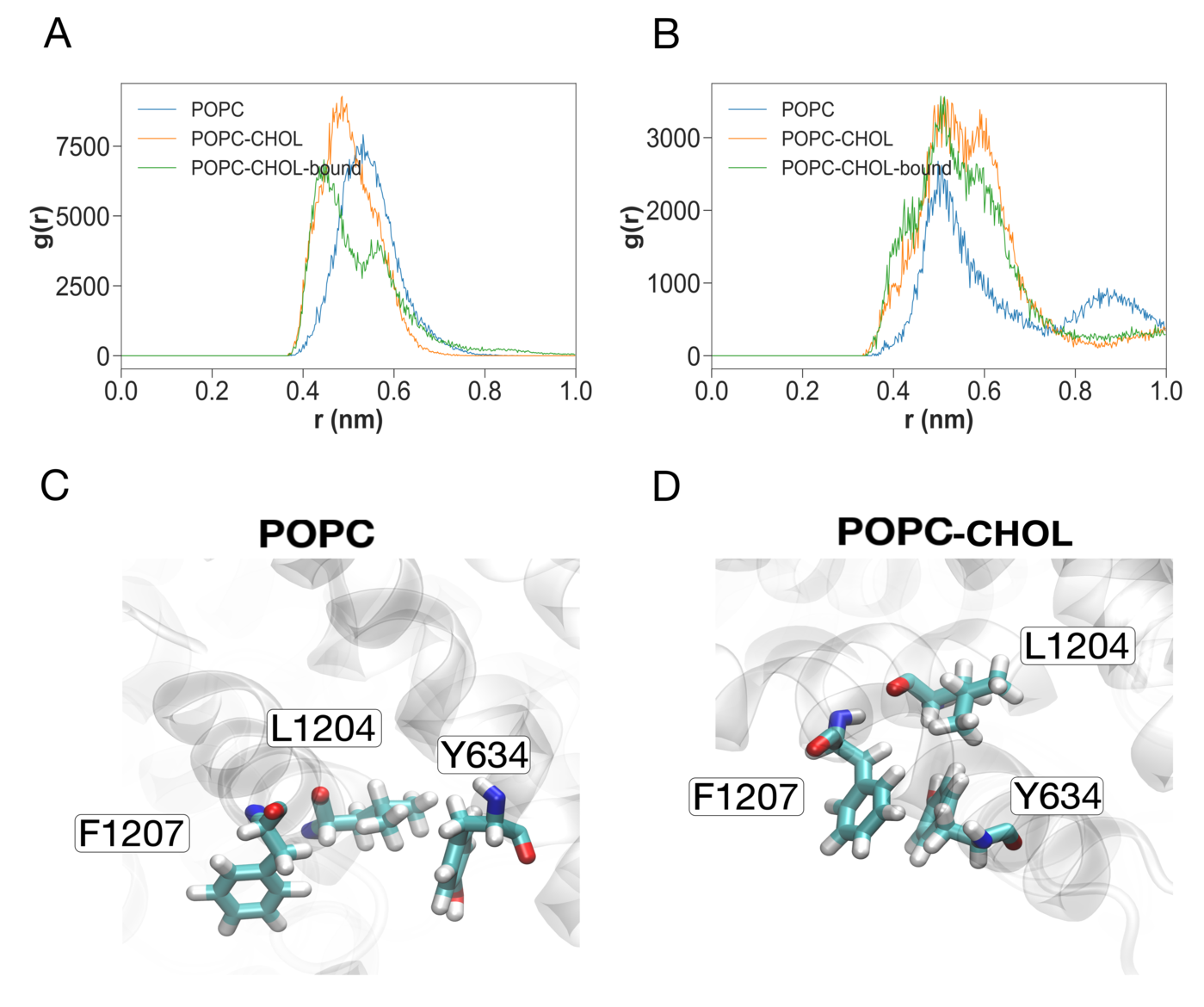

Supplement: S9 Fig — (A) Radial distribution functions between Tyr634 and Leu1204. (B) Radial distribution functions between Tyr634 and Phe1207 (C) and (D) Simulation snapshots from the POPC and POPC-CHOL systems respectively. (TIF) [file pcbi.1007554.s009.tif]

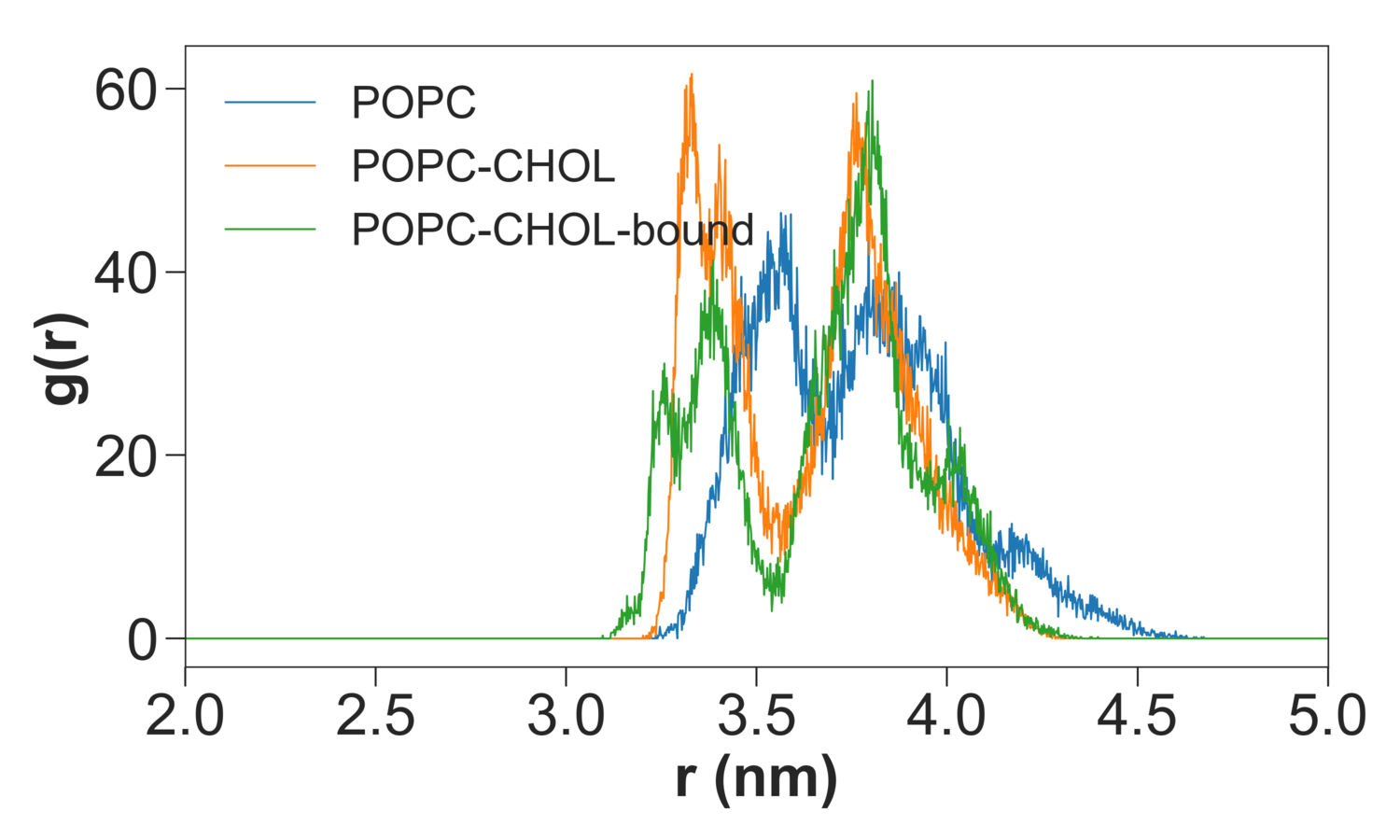

Supplement: S10 Fig — The two domains move apart in the POPC simulations on an average, owing to the flexing of the NTD towards the membrane. (TIF) [file pcbi.1007554.s010.tif]

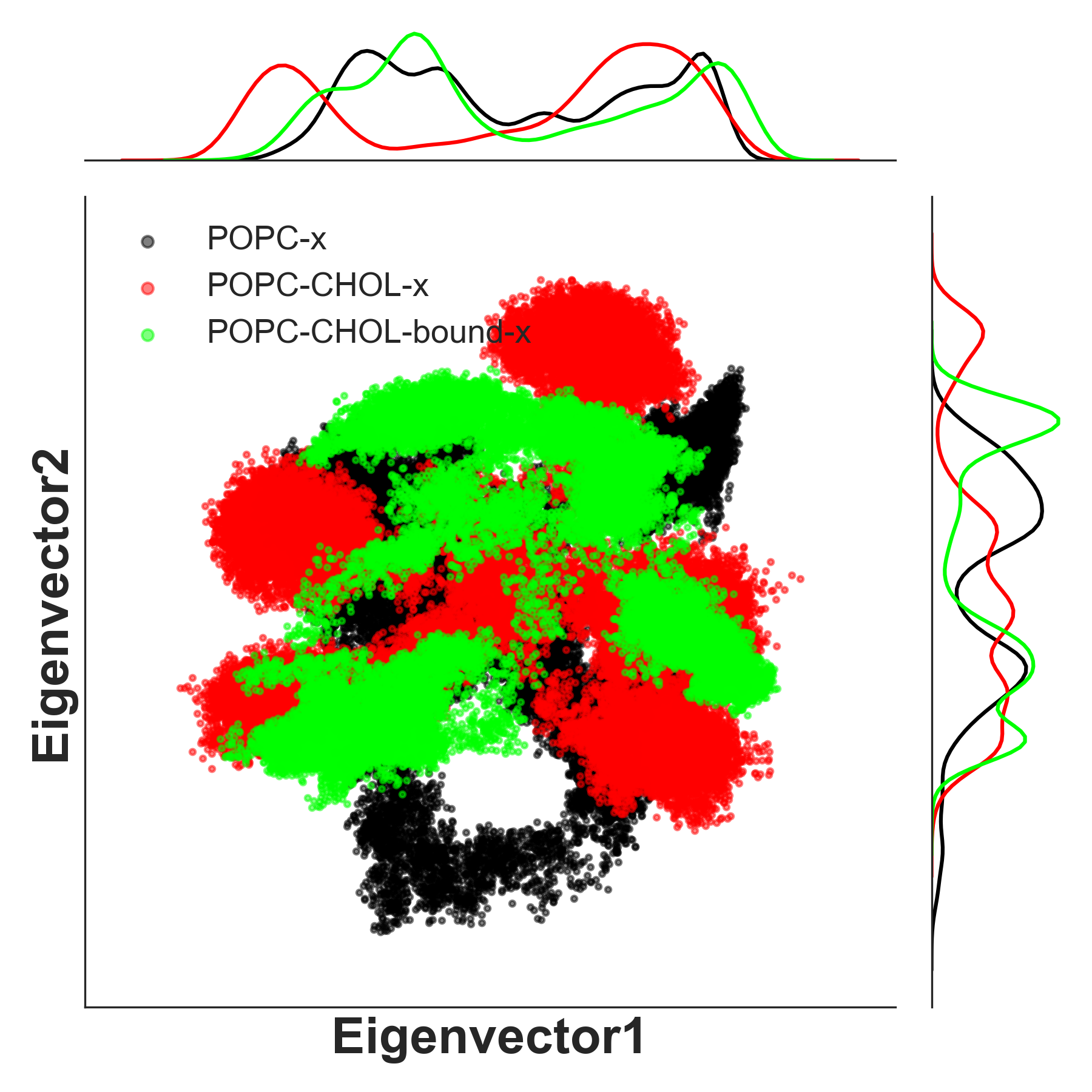

Supplement: S11 Fig — Scatter plot of the all trajectory frames projected on first two eigenvectors of the POPC-x simulation. The analysis is performed on concatenated trajectories of 5 independent 200 ns simulations in each case. (TIF) [file pcbi.1007554.s011.tif]

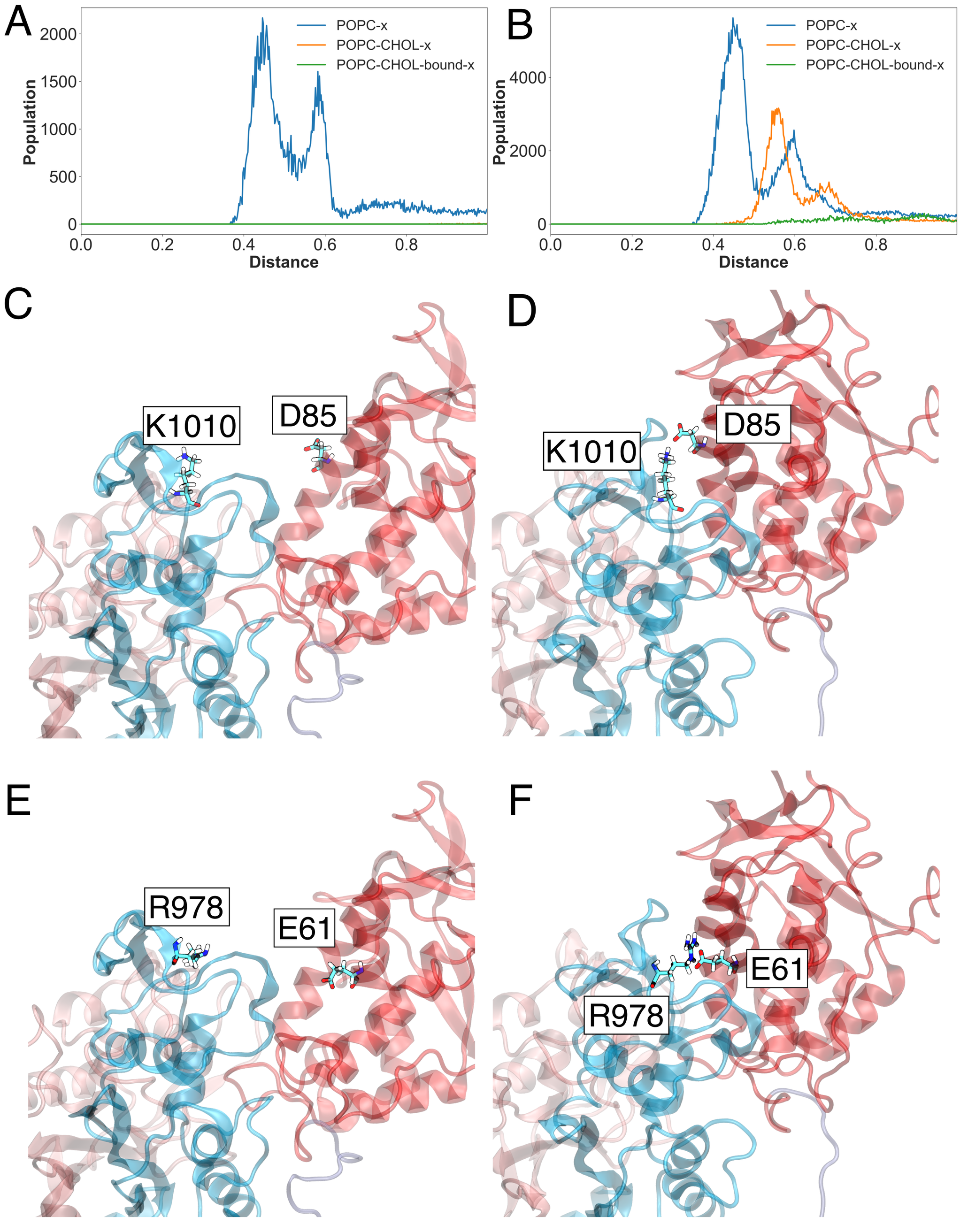

Supplement: S12 Fig — The salt-bridges between the NTD and the CTD in the POPC-x simulations: (A) Radial distribution function between the center of masses of Asp85 and Lys1010. The salt bridge only exists in the POPC-x simulations (B) Radial distribution function between the center of masses of Glu61 and Arg978. The salt bridge is much stronger for the POPC-x simulations (C) and (D) Initial and final POPC-x simulation snapshots corresponding to the interactions in A. (E) and (F) Initial and final POPC-x simulation snapshots corresponding to the interactions in B. The analysis is performed on concatenated trajectories of 5 independent 200 ns simulations. (TIF) [file pcbi.1007554.s012.tif]
