# Supplementary material for: Cholesterol binding to the sterol-sensing region of Niemann Pick C1 protein confines dynamics of its N-terminal domain
Source: PLoS Comput Biol. 2020 Oct 6;16(10):e1007554. doi: 10.1371/journal.pcbi.1007554 (PMC7537887; doi:10.1371/journal.pcbi.1007554)
Supplement: Attachment — Submitted filename: response_to_referees.pdf [file pcbi.1007554.s015.pdf]

We thank both the referees for their very insightful comments. Addressing them required us to run new simulations and analysis, and this has resulted in, what we perceive as a much-improved manuscript. Furthermore, we have revamped the discussion in light of recent developments in the field which point towards the existence of a tunnel through which cholesterol can be transferred form the NTD to the SSD.

Below, our responses to the individual referee comments (in red) are described in normal black text.

Reviewer #1: Dubey et al. describe an extensive atomistic MD simulation study of the Niemann Pick C1 (NPC1) membrane protein in a pure POPC bilayer, in POPC-Cholesterol mixture, and in pure POPC with a cholesterol molecule bound to the sterol sensing domain (SSD) within the membrane (each 10 simulations of 200ns). In three of 10 simulations without cholesterol, a slight tilting of the luminal N-terminal domain towards the membrane was observed. This observation of a cholesterol-dependent degree of conformational flexibility of the NTD is hypothesized to display an allosteric effect of cholesterol.

Overall, the simulations are state-of-the-art. However, the statistical significance of the observation remains unclear.

1. Also, it is unclear how the small additional tilting of the NTD in absence of cholesterol contributes to the function of NPC1; the NTD is still far away from the membrane surface rendering a direct transfer of cholesterol from the NTD to the membrane unlikely. Additionally, an NTD-bound cholesterol was not even considered in the simulations. Similarly, a second discussed option, a possibly NTD-assisted transport of cholesterol in the initial upright position was not investigated in this study.

We agree that the movement of the NTD in the simulations is not sufficient for a direct transfer of an NTD-bound cholesterol to the membrane. Even if such a transition were to occur in NPC1, we are unlikely to capture it in simulations owing to the limited timescales accessible. However, the focus of the manuscript is the allosteric effect of an SSD-bound cholesterol on the dynamics of the NTD, and this is markedly different in simulations with an SSD-bound cholesterol, versus the simulations without an SSD-bound cholesterol. To validate this observation, we have carried out additional statistical assessments of the angle distributions that we calculated from the MD trajectories. These distributions can be seen as experimental distribution functions, which we deconvoluted using three Gaussian functions (either by fitting or using a Gaussian mixture model) applied to each case. We found that all distributions consisted to three populations, or clusters, but only in the simulation of NPC1 in a POPC membrane without cholesterol, the third population was well-separated from the others (Please see Supplementary Figure 3). These results support that the movement of the NTD is very different in this case.

We thank the referee particularly for the comment about the NTD-bound cholesterol. We performed 5 simulations of 200ns each with an NTD-bound cholesterol for each of the three cases (POPC, POPC-CHOL-bound, POPC-CHOL). This amounts to a total of 3 µs of simulation. The cholesterol molecule remains tightly bound to the NTD in all the new simulations.

The key results are described in a new subsection in the Results, section and in Fig. 10 and Supplementary Figures 11 and 12. We find that the downward tilt of the NTD towards the membrane in the POPC case is suppressed in the presence of an NTD-bound cholesterol. For POPC, the cholesterol-loaded NTD comes in close contact with the CTD. In this case, two salt bridges mediate the interactions between the NTD and CTD (Supplementary Figure 12 in the revised manuscript). Such NTD-CTD interactions do not occur when a cholesterol molecule is already present in both the NTD and the SSD. We postulate that the SSD-bound cholesterol influences the dynamics of the NTD differently when the NTD is loaded with cholesterol. In the current manuscript, we have investigated the conformational dynamics of the "apo" state of NPC1 when the NTD is ready to receive a cholesterol molecule from NPC2. The dynamics of the protein will be different in the "holo" state when the NTD is ligand-loaded, and although this difference is captured in the new simulations, a detailed discussion of the allosteric possibilities when cholesterol is present both in the SSD and the NTD is beyond the scope of the current manuscript. We hope to address this issue in future work.

We have elaborated upon the possible NTD-assisted transport of cholesterol in the initial upright position in the discussion section.

Main points:

2 The simulation structure is based on a composed model of a crystal structure, an additional modeled transmembrane helix and NTD (from cryo-EM). The stability of the structure during the equilibration was hardly addressed in the manuscript. However, also the cholesterol-bound simulations show substantial deviations from the initial structure (rmsd plots in Fig. S1).

It is correct, that the entire protein shows substantial fluctuations under all circumstances, which is expected due to thermal fluctuations at room temperature compared to the 'frozen' start structures obtained from X-ray diffraction and cryo EM. What we find, though, is that the accessible conformational space is different depending on cholesterol binding to the SSD.

We observed no major unfolding events in the modeled structures. The starting structure used in the simulations is very similar to the recently published cryo-EM structure (PDB id: 6UOX). The observed RMSD between the modelled structure and the cryo-EM structure is 1.45 Å.

During the simulations, the RMSD deviations from the initial structure are high,

because the luminal domains are dynamic, in particular the NTD. Owing to our careful equilibration protocol in the simulations, we are confident that the deviations from the initial structure faithfully represent the dynamics of the protein, rather than possible simulation artifacts.

The high RMSD of the cholesterol-bound simulations also arises from the dynamics of the luminal domains. The figure below shows the root mean squared fluctuations for the protein for one of the POPC-CHOL-bound simulations. In this simulation, for example, the NTD and CTD have high fluctuations, which contribute to the high overall RMSD.

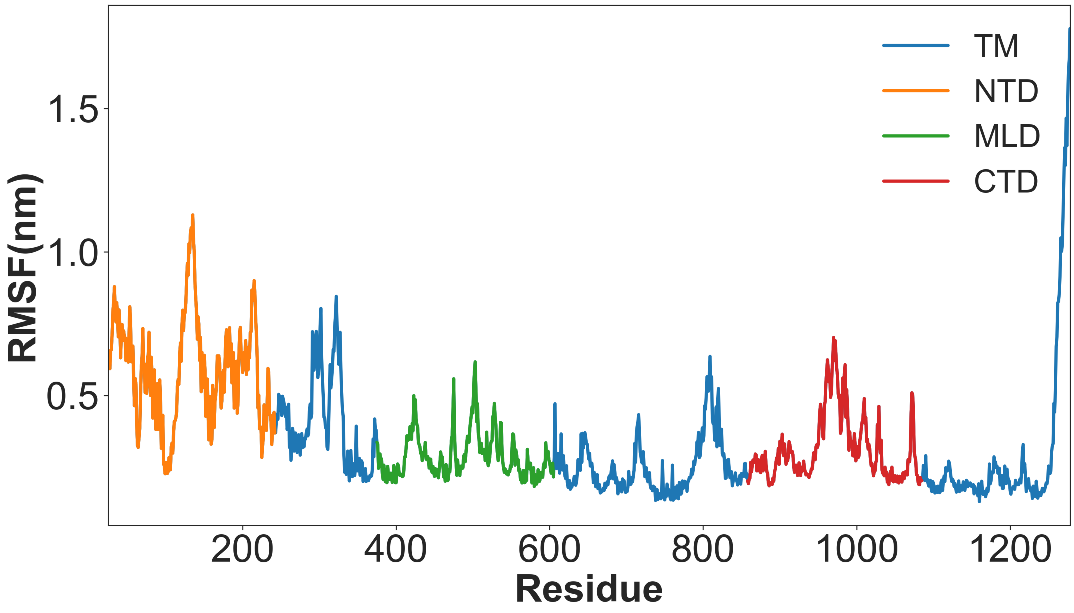

3. In three out of ten simulations an increased tilting of the NBD towards the membrane was observed. For which percentage of overall simulation time was this observed? Fig.4A suggests that this was the case for a very limited time, only. Also, after tilting towards the membrane, did the NTD swivel back during simulation?

We are uncertain if the referee is referring to Figure 3A or Figure 4A. All 10 POPC trajectories were used to calculate the angle distributions shown in Figure 3A. Since the tilting of the NTD was visually explicit in 3 out of 10 simulations, the relative population of the non-tilted upright state is higher compared to the tilted state, giving the impression that the tilting occurs only for a limited time. If conformations corresponding to an α value of 60 degrees of higher are marked as tilted, then the percentage of time for which the NTD is tilted is 44.8%, 24.5% and 28.7% for the POPC, POPC-CHOL and POPC-CHOL-bound simulations respectively. The percentage is slightly higher for POPC-CHOL-bound compared to POPC-CHOL, because in replicate number 4 of the POPC-CHOL-bound system, the cholesterol molecule escapes from the SSD and the NTD bends, please see response to comment 4 of the second referee below, Supplementary Figure 1, and the revised text on page 4. The figure below shows the distribution of α for only the three trajectories where the tilting was observed. If analyzing only these three simulation copies, the percentage

of the time during which the NTD is tilted increases to 82.3 %. Note that this definition of the tilted state is somewhat arbitrary and depends on the choice of the cutoff α. We chose 60 degrees because it corresponds to the first peak in the α distribution of Figure 3. Because the choice of α is somewhat arbitrary, we have not included this information in the revised manuscript. We contend instead that the distributions shown in Figure 3 are more representative of the overall conformational distribution of the tilted and non-tilted states.

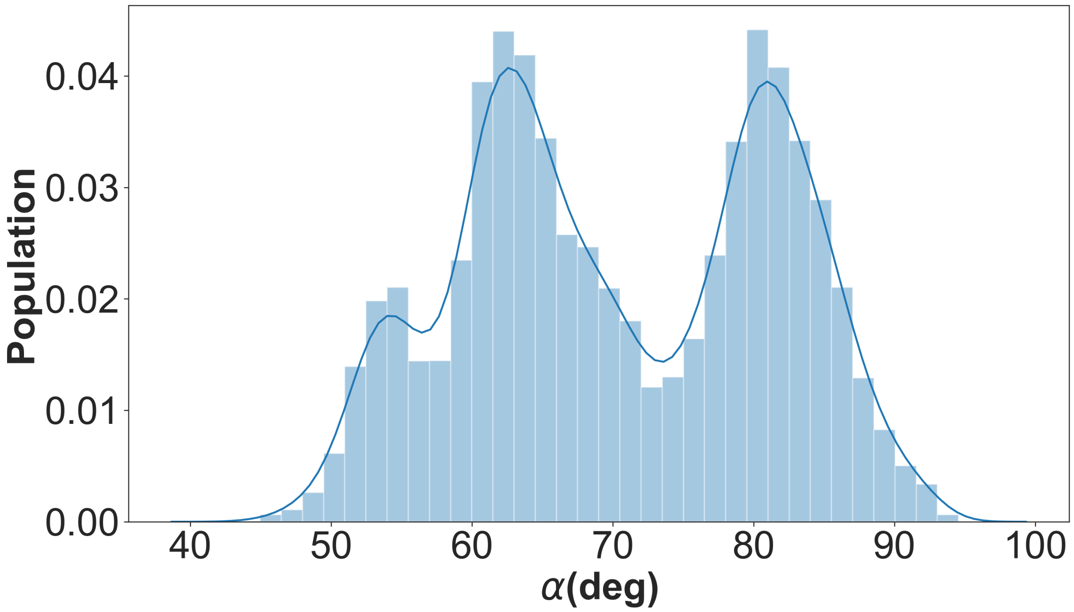

The NTD does not swivel back to the original upright state. This has been noted in the manuscript.

4. p5: The authors claim that "the NTD distance for POPC-CHOL appears to be about 1 nm greater compared to POPC in the distance distribution (Figure 3A)" and that "the distance between NTD and membrane is significantly higher" for the POPC-CHOL-bound simulation as compared to POPC-CHOL. These findings can hardly be seen in Fig. 3. With membrane cholesterol the distance distribution may be narrowed, but a shift is not visible.

Thank you for pointing this out. We have now described the data in Figure 3A more accurately. For the first comment, we have replaced “the NTD distance for POPC-CHOL appears to be about 1 nm greater compared to POPC in the distance distribution” by “the distance distribution for POPC-CHOL is shifted to higher values by about 1 nm compared to POPC”

Secondly, in the last sentence, we were referring to differences between POPC and POPC-CHOL, and not differences between POPC-CHOL and POPC-CHOL-bound. We have replaced “the distance between NTD and membrane is significantly higher” by “the distance between the NTD and the membrane is significantly higher for POPC-CHOL compared to POPC”

5. It would be great if the authors would include a scheme displaying the conformational transitions along the computed eigenvectors.

The conformational transitions along the computer eigenvectors are shown next to the axes in Figure 4. Since these snapshots are small, we also included an animation in the online version that clearly displays the conformational transitions along the computer eigenvectors.

6. p11, statistics: "Many of the amino acid residues belonging to the fragments implicated in the allosteric 289 path are conserved across Eukarya." How many of them are conserved (%)? If randomly choosing fragments, how large is the conservation then?

The average conservation in the five fragments of the allosteric path is (50 + 50 + 0 + 25 + 50)/5 = 35%. The referee is correct in pointing out that the average conservation of the fragments in the allosteric path is not higher than the overall sequence conservation, even though the conservation in the allosteric path is higher than conservation in randomly chosen fragments. We randomly chose 100 fragments using python's random package and estimated the sequence conservation in these fragments. The average percentage conservation is in a randomly chosen fragment is 16.5%. We have therefore removed this sentence from the manuscript, as well as the sequence alignments from the SI. The overall % sequence conservation ranges between 30-90% between different organisms.

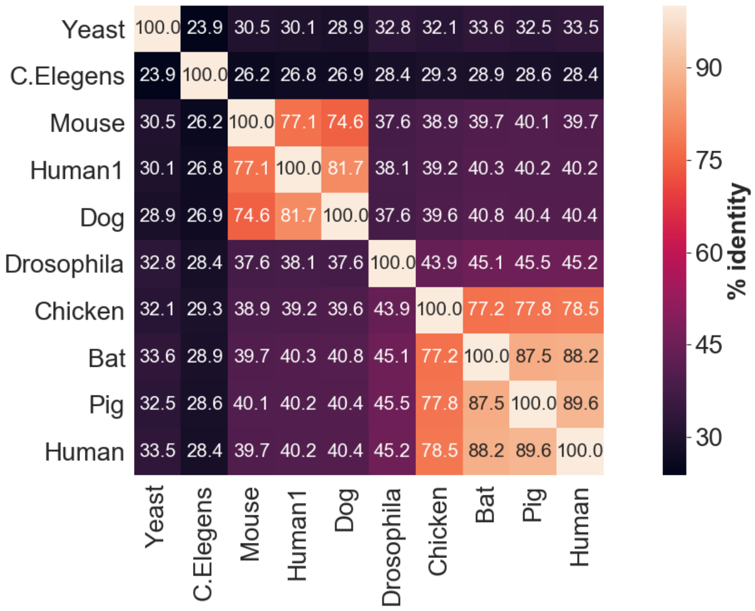

Figure 1: The overall sequence identity of NPC1 across various organisms

7. The authors discuss in detail the impact of bound cholesterol on residue-residue interactions. This is done by comparing distances from three (out of ten) simulations with tilted NBD domain (without cholesterol to all simulations cholesterol. Many interactions are declared to be weakened and discussed. However, an overall picture

is lacking how binding of cholesterol restricts the conformational space of NBD. I.e., what is the sequence of events? Which one is the decisive step, why is a small tilting only observed in three of the ten simulations?

Figure 10 of the original manuscript provided the network of events involved in the flexing of the NTD. We have rewritten the discussion to stress upon the possible sequence of events. "*In the presence of cholesterol, a salt bridge between Asp620 and Lys1217 stabilizes the upright conformation by the network of interactions between the luminal domains as described above. In the absence of cholesterol, the salt bridge breaks, resulting in the unfolding of TM3, leading to the severing of MLD-CTD contacts, in turn severing contacts between the CTD and the NTD. In this way, the NTD is released from the rest of the luminal domains, and approaches the membrane in some of the POPC simulations. The binding of cholesterol in the SSD, and the consequent stabilization of the Asp620-Lys1217 salt bridge has a profound impact on NTD conformational dynamics*." In spite of the importance of the Asp620-Lys1217 salt bridge, we will prefer to stop short of calling this a decisive step, because based on our data, the function of the protein is driven by a complex network of interactions between many residues and between the luminal domains, and interfering with one or many of them can restrict or relieve the conformational freedom of the NTD.

The tilting of the NTD, and its release from the rest of the luminal domains is a stochastic process. In the absence of cholesterol, NPC1 most likely samples a large number of conformational states, and in some of these, the NTD is tilted towards the membrane. As described above, the tilting is statistically significant, and is representative of the conformational freedom of the NTD in the absence of an SSD- and NTD-bound cholesterol. Perhaps we would observe several more of these tilting "events" in longer simulations, but our data clearly show that the NTD has significantly more conformational freedom in the absence of an SSD-bound cholesterol.

Concerning a potential decisive step; we doubt very much that there is one 'committed step' to which textbook biochemistry knowledge often relates. This has been shown long time ago for metabolic pathways in the form of metabolic control theory, identifying the distribution of control or sensitivity over many reaction steps adding up to the regulation of an entire pathway. Similarly, current models of allostery rather emphasize the dynamic nature of many small conformational changes which add up to altered dynamics of a conformational ensemble of a protein upon binding of an allosteric ligand. In the discussion (already in the original form of the manuscript), we advocate this ensemble view of allostery (refs. 79-82 in the revised version) according to which ligands can regulate protein function allosterically without gross conformational changes.

Minor points:

p5, line 170: Fig. 3A should be Fig. 3D

We have corrected this in the revised manuscript.

Figure 3 is not fully displayed in manuscript
We have corrected this in the revised manuscript.

Fig. 4B, D different lines should be explained in the caption

The different lines are now explained in the caption.
"The solid and dashed lines represent the first two orthogonal eigenvectors, projected upon the eigen-space of the POPC systems"

p10-15. The results section suffers from the repeated discussion of numbered fragments that are difficult to relate to structure; It is probably easier to directly name segments by residue numbers.

Thank you for the comment. The avoid the confusion between fragments and residues, we have added a table describing the definition of each fragment observed in the allosteric path.

Reviewer #2: In this work Dubey et al. study how Cholesterol binding to the sterol-sensing region of a Niemann Pick C1 protein confines the motion its N-terminal domain. The work's take home message is rather striking and inspiring: Binding of a single cholesterol molecule to a distal cluster of transmembrane domains can trigger a whole allosteric 'chain reaction' that restricts large conformational changes of a distal n-terminal domain. Furthermore, the resolved allosteric mechanism/pathway is extensively compared to known putative point mutations and conserved residues. I believe these insights to be highly valuable for the field of structural biology since it can causally explain the relationships between them. Finally, the performed simulations and analysis are both solid and state-of-the-art. I have only few minor comments on this work:

1. Page 5 in the title: .....cholesterol-dependant -> dependent

Thank you for highlighting the spelling error. We have corrected it

2. Page 8: "and the rate constant for binding is bimolecular."
I do not follow the flow of logic or implication of this statement. Yes. There are only two molecular species, but why one rate constant? Is the binding competitive? Are lipids able to bind into that pocket as well, however, with a far weaker affinity. Hence, cholesterol is not knocked out from the binding pocket in the cholesterol free simulation.

Since binding involves two molecules, a protein and a ligand, the binding reaction is second order, hence the rate constant for binding (k_{on}) is bimolecular, i.e., it depends on the concentration of the ligand with unit mole/sec. Accordingly, increasing the abundance of the ligand cholesterol in the membrane will speed up the binding process (increase k_{on}). The same is not true for the release rate constant, k_{off} which is monomolecular with units 1/sec. Accordingly, increasing cholesterol in the membrane will greatly influence the binding rate without affecting cholesterol release with the net effect that cholesterol will always be SSD-bound in a cholesterol enriched lysosomal membrane. The fact that the binding rate constant is bimolecular has thereby a large effect on the overall kinetics of the allosteric regulation, we have discovered.

We have made this clarification in the revised manuscript.

3. Page 8: "We simulated an extra system with a different cholesterol distribution in the membrane, and in this case too, cholesterol bound to the SSD on a timescale of" Distribution -> Composition? Note that this extra system is also nowhere described.

Thank you for comment. We would like to clarify that we meant to use word 'distribution'. For the long simulation, we positioned cholesterols in such a way that they were not near the SSD-binding site. Therefore, we changed the initial positions of cholesterol on the membrane. We have corrected the description in the revised manuscript. The composition of the cholesterol was kept at 20% (similar to the POPC-CHOL case). The goal of the additional simulation was to check the impact of a different positioning of cholesterol in the membrane. In addition, given that we already have 10 simulations of the POPC-CHOL system, we decided not to include the 11th system for consistency. All other simulations systems have 10 replicates.

4. Page 8: Does cholesterol ever unbind? Worth looking at how long cholesterol stays bound in the POPC-CHOL-bound system?
Perhaps add an explicit statement about this since the reader will be wondered about this.

This was certainly worth looking at, and we thank the reviewer for this suggestion.. We observed that cholesterol does unbind and rebind in some of the POPC-CHOL-bound simulations. We have described this equilibrium in the revised manuscript, and in Supplementary Figure 1 (copied below) The figure measures the distance between SSD bound-cholesterol and center of mass of the residues in the binding site (as shown in Fig 1A).

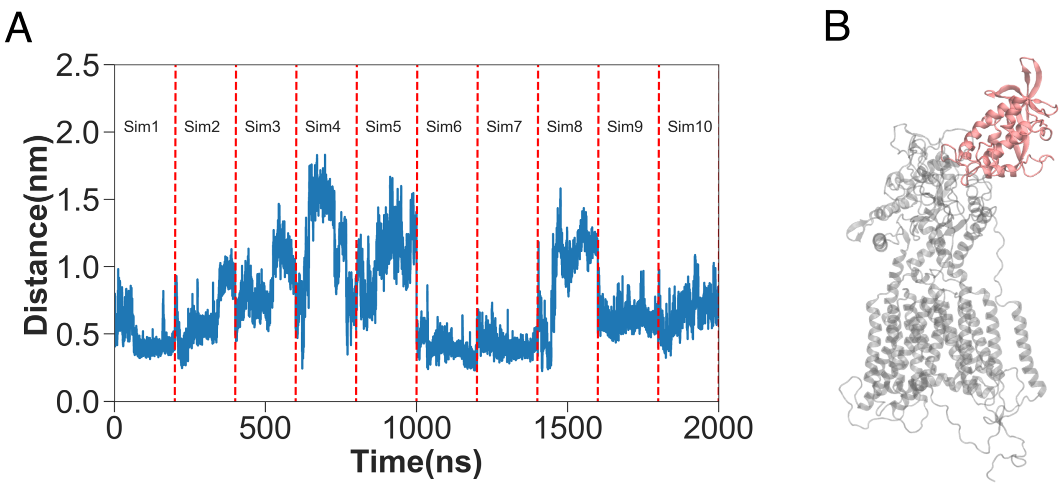

From the figure, we can conclude that the cholesterol temporarily escapes the binding pocket in sims 3,4,5 and 8. Remarkably, in sim4, in which cholesterol is farthest away from the SSD, the NTD does adopt a tilted orientation, confirming further that the presence of cholesterol in the SSD is correlated to the tilting of the NTD.

Page 12: Fig.4. Description of figure E is missing within the caption.

Thank you for pointing this out. We have added this description

Inconsistent use of capital letters throughout the manuscript. For example:
Page 8: "Covariance and principal components Analysis" Check capital letters.
Thank you for pointing this out. We have corrected the mistake in the manuscript.

Page 9: "between two vectors: one vector" -> :One vector (capital)

Thank you for pointing this out. We have corrected the mistake in the manuscript.

Page 10: Fig 2 -> "Representative Initial" -> .....initial

Thank you for pointing this out. We have corrected the mistake in the manuscript.

Page 11: origin in Figure 4A and Figure 4C. Check whether the F in 'Figure' must capitalized when not being an abbreviation. One page later, on page 15, l296: "Supplementary figure" ...figure is written with a small "f"

Thank you for pointing this out. We are now more consistent with the capitalization. All Figure citations are now “Fig.” instead of “Figure” or “figure”.
